# Vaccination-enabled immune readiness for checkpoint blockade

**DOI:** 10.3389/fimmu.2026.1790344

**Published:** 2026-07-08

**Authors:** Jhommara Bautista, Andrés López-Cortés

**Affiliations:** Cancer Research Group (CRG), Faculty of Medicine, Universidad de Las Américas, Quito, Ecuador

**Keywords:** cancer therapy, immune checkpoint blockade, immune readiness, primary resistance, therapeutic vaccination

## Abstract

Immune checkpoint blockade has transformed cancer therapy by demonstrating that durable tumour control can be achieved through immune modulation rather than direct cytotoxicity. However, primary resistance remains the dominant clinical outcome across solid tumours, reflecting a fundamental limitation of checkpoint inhibitors: they do not initiate antitumour immunity, but amplify immune responses that are already underway. Here, we advance immune readiness as a unifying framework to explain primary resistance to immune checkpoint inhibitors. Immune readiness is defined as a dynamic and programmable host–tumour state characterised by competent innate sensing, type I interferon–driven myeloid activation, dendritic-cell licensing, coordinated antigen presentation, productive lymphocyte priming, and permissive inflammatory trafficking into tumour tissue. In the absence of these upstream processes, checkpoint blockade is biologically inconsequential, regardless of tumour antigenicity or checkpoint expression. Within this framework, therapeutic vaccination is positioned as a flexible immune-conditioning strategy that can induce, amplify, or synchronise immune readiness rather than as a direct cytotoxic modality or rigidly antecedent intervention. Tumour-directed, immune-modulatory, and tumour-agnostic vaccines may construct the immunological substrate required for checkpoint efficacy when integrated before, during, or in close temporal coordination with checkpoint blockade. Engineering immune readiness through vaccination-enabled immunotherapy offers a coherent strategy to overcome primary resistance and expand the therapeutic reach of cancer immunotherapy.

## Introduction

Immune checkpoint blockade has transformed the therapeutic landscape of oncology by establishing that durable tumor control can be achieved through immune modulation rather than direct cytotoxic targeting (1, 2). Antibodies against PD-1, PD-L1, and CTLA-4 have produced unprecedented long-term survival in subsets of patients across multiple cancer types, validating the immune system as a clinically actionable anticancer effector. Yet this success is accompanied by a sobering reality: for the majority of patients, immune checkpoint inhibitors (ICIs) fail as monotherapy, with primary resistance remaining the dominant clinical outcome across solid tumors (3, 4).

This widespread lack of response is commonly framed in tumor-centric terms. Non-responsiveness is often attributed to insufficient tumor antigenicity, low tumor mutational burden (TMB), lack of PD-L1 expression, or absence of T cell infiltration, leading to the classification of “cold tumors” as intrinsically refractory to immunotherapy (5, 6). While these associations are statistically robust, they are frequently misinterpreted as causal explanations. Such interpretations risk conflating descriptive biomarkers with underlying biological mechanisms and obscuring a more fundamental limitation of checkpoint blockade: ICIs do not initiate antitumor immunity, but instead act on immune responses that are already underway (7–9).

A growing body of clinical, transcriptional, and mechanistic evidence indicates that the efficacy of checkpoint inhibition is conditioned primarily by the pre-treatment immunological state of the host–tumor system (10, 11). In this view, primary resistance does not reflect failure of checkpoint targeting per se, but rather the absence of the upstream immune processes required to render checkpoint pathways functionally relevant (4, 7). Tumors that fail to respond to ICIs are often embedded within an immune environment that has not achieved the threshold of activation, antigen presentation, and effector deployment necessary for productive immune engagement. Checkpoint blockade applied to such systems is therefore biologically underpowered, regardless of tumor antigenicity or checkpoint expression (8, 12).

This review advances the concept of immune readiness as a unifying framework for understanding primary resistance to immune checkpoint blockade. Immune readiness refers to a dynamic, programmable host state defined by competent innate immune sensing, functional antigen presentation, effective dendritic cell (DC) licensing, coordinated lymphocyte priming, and permissive inflammatory trafficking into tumor tissue (7, 11). From this perspective, resistance emerges not as a fixed tumor-intrinsic property, but as a consequence of treating an immunologically unprepared system. We synthesize mechanistic and clinical evidence demonstrating how failures in immune initiation, rather than excessive immune suppression, underlie most cases of primary resistance (12, 13).

**Figure 1:**
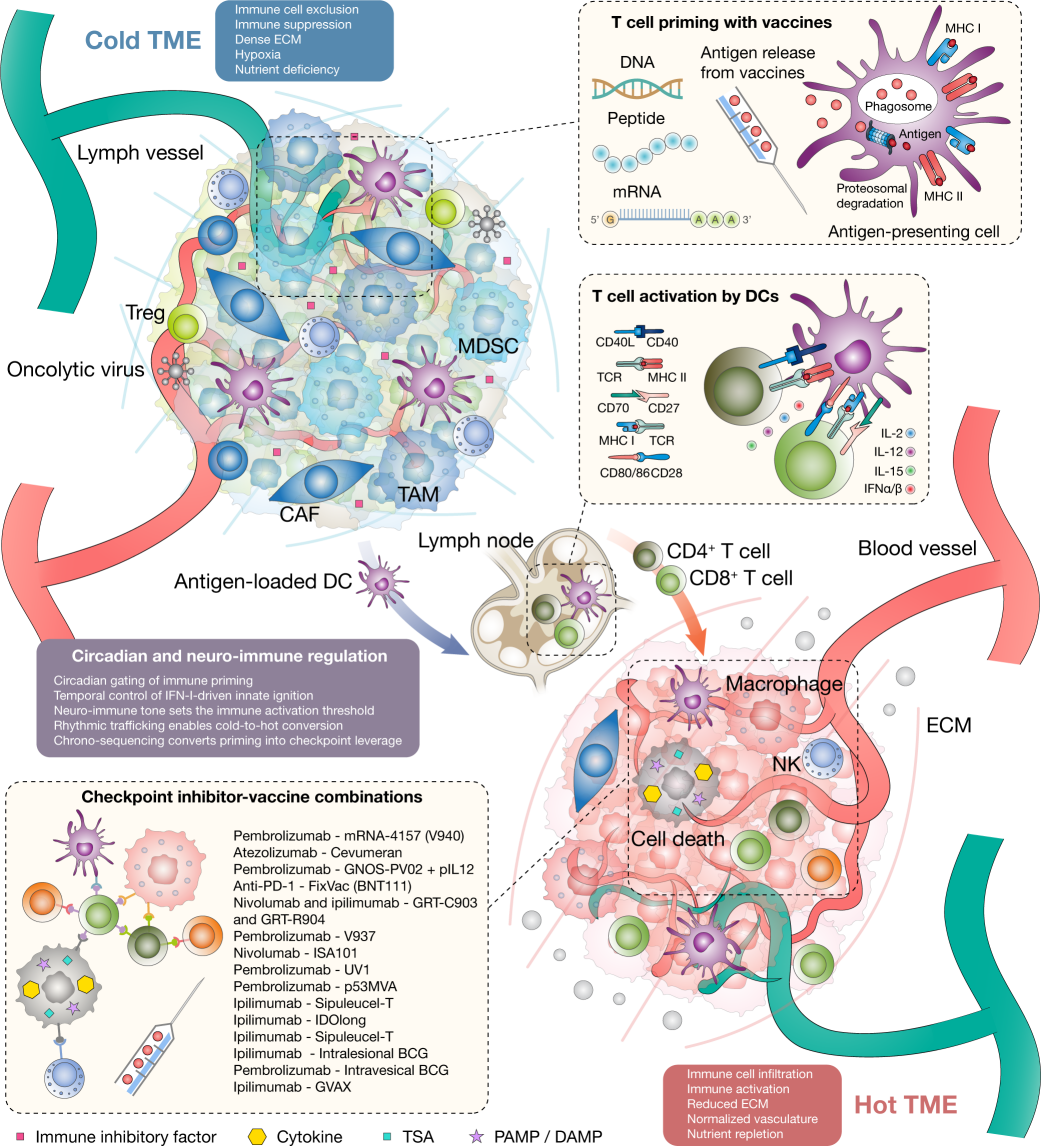
Vaccination-driven temporal priming converts cold tumors into hot tumors and unlocks checkpoint blockade. Schematic overview of how temporal immuno-engineering, shaped by circadian and neuro-immune regulation, governs the conversion of immunologically cold tumors into hot tumors and determines the biological relevance of immune checkpoint blockade. Cold tumors are characterized by immune cell exclusion, dominant immunosuppressive signaling, dense extracellular matrix (ECM), hypoxia, and nutrient deprivation, collectively limiting antigen presentation, lymph-node priming, and effector T cell deployment. Under these conditions, immune checkpoint inhibitors (ICIs) administered in isolation or outside the priming window frequently fail to elicit durable antitumor immunity. Upstream immune priming, achieved through vaccination or other innate-activating interventions, induces type I interferon (IFN-I)–driven innate reprogramming, licensing myeloid cells, restoring antigen presentation, and enabling effective lymph-node priming. This process is temporally gated by circadian rhythms and modulated by neuro-immune tone, which together set the threshold for immune activation. Rhythmic trafficking of immune cells and time-restricted innate ignition facilitate cold-to-hot tumor conversion, yielding a hot tumor state defined by immune cell infiltration, immune activation, reduced ECM constraints, normalized vasculature, and nutrient repletion. Within this reprogrammed context, chrono-sequenced checkpoint blockade, delivered concurrently with or following the priming phase, amplifies effector function, preserves progenitor-like CD8^+^ T cell subsets, promotes epitope spreading, and sustains tumor control. Representative checkpoint inhibitor–vaccine combinations currently under clinical evaluation, including pembrolizumab with mRNA-4157 (V940), atezolizumab with cevumeran, anti-PD-1 with FixVac (BNT111), nivolumab with ISA101, pembrolizumab with UV1, and multiple combinations incorporating GVAX, BCG, Sipuleucel-T, IDOlong, or personalized neoantigen platforms, illustrate how vaccination-enabled or priming-aligned strategies operationalize this temporal logic in patients. Lastly, this framework highlights that sequence and timing are mechanistic variables, not technical details: effective immunotherapy requires circadian- and neuro-immune–aligned priming to construct the hot tumor state upon which immune checkpoint inhibition can act productively.

Building on this framework, we examine vaccination-based strategies as tools to engineer immune readiness without implying a rigid clinical sequence in which vaccination must always precede checkpoint blockade. Rather than using vaccination as a strictly antecedent intervention, we position therapeutic vaccination as a flexible immune-conditioning strategy that can induce, amplify, or synchronise the innate and adaptive immune processes required for checkpoint efficacy. Tumor-directed, immune-modulatory, and tumor-agnostic vaccines can reprogram immune circuits, reshape temporal and spatial dynamics of activation, and help convert immunologically inert tumors into checkpoint-responsive systems (14, 15). Depending on the vaccine platform, tumor context, and therapeutic objective, this immune-conditioning effect may occur before, during, or in close temporal coordination with checkpoint inhibition. Therefore, the central principle is not that vaccines must always come first chronologically, but that immune priming, antigen-presentation competence, and inflammatory trafficking must be established within the therapeutic window in which checkpoint blockade is expected to act. Finally, we integrate emerging insights into type I interferon (IFN-I) signalling, therapeutic sequencing, circadian and neuro-immune regulation, and clinical trial design to outline principles for next-generation immunotherapy strategies that treat immune readiness itself as a therapeutic target (**Figure 1**).

## Immunological determinants of primary resistance to checkpoint blockade

ICIs have established that durable tumor control can be achieved by releasing inhibitory pathways that restrain antitumor T cell activity. However, across cancer types, primary resistance remains the predominant clinical outcome, with the majority of patients failing to benefit from PD-1 or PD-L1 blockade monotherapy (2, 4, 6, 7). This failure is often interpreted as evidence that immune checkpoints are irrelevant in these tumors. A closer examination of tumor–immune biology, however, leads to a different conclusion: in most non-responsive cancers, checkpoint blockade fails not because inhibitory pathways are absent or non-functional, but because the immune system was never brought into a state in which checkpoint release is biologically meaningful (7, 12).

Checkpoint inhibitors target a specific biological substrate: antigen-experienced T cells that have undergone effective priming, clonal expansion, and tissue recruitment. They do not initiate immune responses *de novo*, nor do they compensate for failures in upstream immune activation (6, 12, 13). Clinical and transcriptional analyses across tumor types consistently demonstrate that response to PD-1 blockade is strongly associated with baseline features of immune engagement, including IFN-γ–responsive gene programs, intact antigen presentation machinery, and chemokine expression profiles compatible with effector T cell recruitment (10, 16, 17). Tumors lacking these features typically exhibit sparse or absent intratumoral CD8^+^ T cells, low expression of CXCL9 and CXCL10, and minimal activation of adaptive immune circuits. In such contexts, checkpoint inhibition has little biological leverage, as there is no pre-existing immune response to unleash (6).

A central determinant of this immune deficit is DC dysfunction. Effective antitumor immunity requires cross-presentation of tumor antigens by specialized DC subsets, particularly Batf3-dependent cDC1s, which are essential for priming tumor-specific CD8^+^ T cells in tumor-draining lymph nodes and for orchestrating their subsequent recruitment into the tumor microenvironment (TME) (6, 12, 13, 18, 19). Non-inflamed tumors are characterized by a profound scarcity of these DCs, resulting in defective antigen priming and failure to establish a productive cancer–immunity cycle. Experimental and human studies further demonstrate that the absence of CD103^+^ DCs leads to insufficient production of CXCL9 and CXCL10, chemokines that are indispensable for CXCR3-dependent effector T cell trafficking (4, 17, 20, 21). Under these conditions, even systemically activated T cells are unable to access tumor sites, rendering checkpoint blockade ineffective despite intact checkpoint targets (12, 16).

Beyond priming defects, many tumors exhibit additional layers of immune exclusion that further constrain checkpoint efficacy. These include aberrant vasculature, stromal barriers, and tumor-intrinsic signalling pathways that actively suppress immune infiltration (16, 22). Such mechanisms result in spatial segregation of immune cells from malignant tissue, reinforcing immune ignorance rather than immune suppression. In these settings, the limiting factor is not excessive inhibitory signalling but a failure of immune system engagement at both the initiation and effector phases of the response (6, 11, 23).

These observations challenge the conventional definition of cold tumors as simply non-inflamed or poorly infiltrated lesions. Integrated frameworks incorporating Immunoscore, tumor gene expression profiling, and large-scale clinical datasets indicate that non-responsiveness to ICIs reflects a broader host–tumor system locked in a state of immune unreadiness, rather than a tumor-intrinsic resistance to immune attack (6, 13). In this view, PD-L1 negativity, low TMB, or absent T cell infiltration are not primary causes of resistance, but downstream consequences of a failure to engage innate and adaptive immune programs (7, 8).

Mechanistic studies further reinforce this interpretation by demonstrating that many cold tumors actively downregulate antigen processing and presentation pathways, including HLA class I molecules and their associated chaperones (4, 17, 24). This limits surface presentation of tumor-associated antigens and neoantigens, thereby preventing immune recognition even when potentially immunogenic targets exist. Such alterations do not merely evade checkpoint-mediated control; they preclude the very immune activation upon which checkpoint blockade depends (7).

Taken together, these findings support a conceptual shift in how primary resistance to ICIs is understood. Rather than reflecting failure of checkpoint inhibition *per se*, resistance most often arises from the absence of immune readiness, a dynamic, multilevel host state encompassing innate immune competence, functional antigen presentation, effective DC licensing, and coordinated lymphocyte trafficking. Immune checkpoints become relevant only once this state has been achieved. Without it, releasing inhibitory signals is biologically inconsequential. In this light, checkpoint blockade fails in cold tumors not because the brakes are missing, but because the engine never started.

## Immune readiness as a dynamic and programmable host state

A unifying lesson from clinical checkpoint blockade is that ICIs do not create antitumor immunity from nothing; rather, they amplify, sustain, or reinvigorate immune responses that are already underway, which immediately shifts the explanatory burden from tumor-intrinsic checkpoint expression to the pre-treatment immunological status of the host–tumor system (3, 7, 9, 25). This is the conceptual core behind the cancer–immune set point: diverse tumor, host, and environmental variables combine to determine whether anticancer immunity crosses the threshold required for clinical response, even among patients with superficially similar tumors (7, 26). In that framing, primary resistance is not simply a property of cold tumors, but the predictable outcome of treating a system that has not achieved the immunological prerequisites for productive checkpoint release (4, 10, 18, 27).

Operationally, immune readiness can be defined as the integrated capacity of the host immune system to (i) sense tumor-derived danger and initiate innate activation, (ii) present antigen effectively through functional antigen presenting cells (APCs), (iii) generate and expand tumor-reactive clones, and (iv) deploy those effectors into tumor tissue within an inflammatory context that remains sufficiently plastic to support cytotoxic function without collapsing into paralysing suppression (4, 7, 26). The first component is the innate competence, where, in immunogenic tumor settings, innate recognition depends on tumor-derived signals that activate host pathways capable of inducing IFN-Is, which in turn license APCs and bridge innate sensing to adaptive priming (27–29). Without this upstream ignition, a tumor can remain immunologically silent, and PD-1/PD-L1 blockade becomes mechanistically underpowered because there is little ongoing effector activity to release (3, 6, 9).

The second component, antigen presentation capacity, centres on cross-presentation by specialized DC lineages. The Batf3-dependent CD8α^+^/cDC1 compartment is a canonical example: Batf3 deficiency ablates development of this lineage, impairs cross-presentation, and compromises cytotoxic T cell immunity and rejection of immunogenic tumors, illustrating that antigen presentation is not a generic function distributed evenly across APCs but a lineage-specialized bottleneck (26, 27, 30). Clinically, this logic maps onto a broader observation emphasized in resistance frameworks: checkpoint inhibitors rely on pre-existing tumor-reactive T cells whose function is restrained by inhibitory pathways, and resistance frequently converges on failure points upstream of checkpoint release, including deficits in priming and antigen presentation (3, 4, 26). In other words, immune readiness is partly the availability of a competent antigen-presentation axis that can continually feed tumor-specific clones into the response (30).

The third component, clonal expansion potential, is a property of both the repertoire and its activation state. The most direct human evidence that baseline adaptive engagement matters comes from biomarker studies showing that response to PD-1/PD-L1 pathway blockade associates with transcriptional signatures of a pre-existing, activated T cell programme in the TME (3, 9). Early clinical biomarker work with anti–PD-L1 (MPDL3280A) found that responses were enriched in patients whose baseline tumors exhibited immune features consistent with suppressed pre-existing immunity, including TH1-associated gene expression, supporting the view that PD-L1 blockade is most effective when it releases an already engaged immune response rather than attempting to initiate one (9). Large pan-tumor analyses further formalize this into inflammatory biomarkers: the T cell–inflamed gene expression profile (GEP) captures a coordinated programme of antigen presentation, chemokine expression, cytotoxic activity, and adaptive immune resistance, and it stratifies response to pembrolizumab across tumor types, partly independently of TMB (6). Similarly, an IFN-γ–related mRNA profile predicts clinical benefit from PD-1 blockade and explicitly links response likelihood to a baseline IFN-responsive state that includes antigen presentation and chemokines required for effector recruitment, while also noting these features are often necessary but not sufficient (10, 31). Together, these datasets support a practical interpretation: immune readiness is observable as a baseline immune contexture enriched for activated T cells and IFN-responsive programmes, which provides the substrate for checkpoint release (7, 10, 25).

The fourth component, inflammatory plasticity, captures the fact that readiness is not simply more inflammation, but the ability to mount and sustain an effective inflammatory trajectory without being diverted into dysfunctional, suppressive, or spatially excluded states. This is why immune profiling studies often identify not only the presence of infiltrates but the specific immune states that matter (7). For example, deep tumor profiling in melanoma has shown that response to anti–PD-1 monotherapy and anti–PD-1/anti–CTLA-4 combination therapy is associated with activated T cell signatures and defined effector memory phenotypes, while non-responders can present “TIL-hot” tumors that nevertheless express alternative inhibitory programmes and targets, indicating that the qualitative state of immunity conditions therapeutic success (4, 9, 10, 25). This nuance aligns with broader immune-evasion models emphasizing that resistance is dynamic and can reflect either adaptive resistance within an inflamed tumor or innate evasion states in which key immune populations are excluded, reinforcing that the relevant unit is the tumor embedded within a host immune system whose state can be permissive or non-permissive (4, 26).

A critical distinction, therefore, is between baseline immune contexture and induced immune readiness. Baseline contexture refers to the pre-treatment configuration (immune infiltrates, antigen presentation competence, and IFN/TH1-associated transcriptional programmes) that correlates with response to PD-1/PD-L1 blockade across studies (9, 31). Induced readiness, by contrast, refers to a state transition in which innate sensing and APC licensing activate, expand, and mobilize tumor-reactive clones such that checkpoint pathways become biologically consequential (28). Mechanistically, the STING–IFN-I axis offers a clean illustration of how such readiness can be initiated upstream of adaptive control, because it links tumor-derived DNA sensing to IFN-β production and DC activation, events that are upstream of T cell priming and hence upstream of where ICIs act (27, 29). Conceptually, the set point framework then explains why similar tumors can diverge clinically: what differs is not only tumor antigenicity but the composite of host-intrinsic and extrinsic factors that place an individual above or below the threshold for effective antitumor immunity (7, 26).

For this framework to become clinically useful, immune readiness must also be operationalized as a measurable state rather than only a conceptual descriptor. We propose that readiness should be assessed through a composite immune-readiness panel integrating tumour-biopsy, peripheral-blood, and, where feasible, tumour-draining lymph-node measurements. No single marker is sufficient to define a checkpoint-responsive state across tumour types; instead, readiness should be inferred when multiple upstream immune modules are simultaneously detectable, including innate activation, antigen-presentation competence, DC licensing, T-cell priming, inflammatory chemokine production, and effector trafficking (6, 10, 15). Conversely, immune unreadiness is suggested by the coordinated absence of these modules despite preserved tumour antigenicity or checkpoint expression.

In routine tumour biopsies, pre-existing readiness may be inferred from a T cell–inflamed GEP, IFN-γ–responsive transcriptional programmes, intact antigen-processing machinery, and chemokine gradients compatible with effector recruitment, particularly CXCL9, CXCL10, and CXCL11 (6, 10). Additional supportive features include intratumoural or invasive-margin CD8^+^ T-cell infiltration, expression of cytotoxic effector genes such as GZMB, PRF1, and IFNG, and preservation of HLA class I, B2M, TAP1/2, and other antigen-processing components (10, 31). By contrast, immune unreadiness is suggested by sparse CD8^+^ T-cell infiltration, low or absent CXCL9/CXCL10 expression, defective HLA class I or antigen-processing machinery, scarce cDC1 signatures, and a non-inflamed or immune-excluded architecture (22, 32).

Because DC licensing is a central bottleneck upstream of checkpoint responsiveness, the abundance and activation state of cross-presenting DCs should be incorporated whenever feasible. In tumour tissue or TDLN aspirates, enrichment of cDC1-associated markers such as XCR1, CLEC9A, BATF3, CD141 in humans, or CD103 in selected translational contexts, together with activation and migratory markers such as CD40, CD80, CD86, CCR7, and HLA-DR, would support antigen-presentation competence (21, 32). Conversely, low cDC1 abundance, poor DC activation, or absence of CCR7-dependent migratory signatures would indicate impaired priming capacity and therefore an unprepared immune state.

Peripheral blood cannot fully substitute for tumour or nodal profiling, but it can help distinguish pre-existing readiness from induced readiness after vaccination or immune conditioning. Pre-existing readiness would be reflected by baseline inflammatory and T-cell activation signals already present before therapy. Induced readiness, by contrast, should be defined as a dynamic increase after vaccination or innate immune stimulation, including expansion of antigen-specific or vaccine-reactive T-cell clones, increased activated or proliferating Ki-67^+^ CD8^+^ T cells, transient IFN-I or IFN-γ–linked transcriptional induction, increased circulating CXCL9/CXCL10, and evidence of T-cell receptor repertoire broadening or epitope spreading (33, 34). Thus, the relevant clinical question is not whether a tumour expresses a single biomarker above a universal threshold, but whether the host–tumour system has crossed a multidimensional immune-activation threshold sufficient for checkpoint blockade to amplify rather than prematurely release an absent or defective response.

In practical terms, an operational immune-readiness panel could include: (i) tumour-biopsy RNA profiling for T cell–inflamed GEP, IFN-γ signatures, antigen-presentation genes, and CXCL9/CXCL10; (ii) multiplex immunohistochemistry or flow cytometry for CD8^+^ T cells, cDC1s, activated DCs, and spatial immune exclusion; (iii) peripheral-blood flow cytometry for activated and proliferating CD8^+^ T cells, myeloid activation, and progenitor/exhaustion-associated markers; (iv) TCR sequencing to detect clonal expansion or repertoire broadening; and (v) optional TDLN aspirate analysis to measure DC licensing and early T-cell priming (6, 10, 33, 34). This framework does not impose a universal numerical cut-off, but provides a clinically testable structure for distinguishing immune-ready, inducibly ready, and immune-unready states. Together, these measurements define a practical operational framework for classifying immune-ready, inducibly ready, and immune-unready states before or during vaccine–checkpoint inhibitor strategies (**Table 1**).

**Table 1:** Proposed operational panel for assessing immune readiness in vaccine–checkpoint inhibitor strategies.

Immune-readiness module	Source	Suggested assays or readouts	Immune-ready or inducibly ready state	Immune-unready state
Tumour inflammatory contexture	Tumour biopsy	RNA-seq, targeted immune transcriptomics, spatial transcriptomics, multiplex IHC/IF	T cell–inflamed GEP; IFN-γ/IFN-I–responsive programmes; inflammatory immune contexture	Immune-desert, immune-excluded, or non-inflamed phenotype
Chemokine-mediated effector trafficking	Tumour biopsy; plasma/serum as supportive readout	CXCL9, CXCL10, CXCL11 by RNA profiling, qPCR, ELISA, or multiplex imaging	Enriched CXCL9/CXCL10/CXCL11 compatible with CXCR3^+^ effector T-cell recruitment	Low or absent CXCL9/CXCL10; weak effector-cell recruitment signals
Antigen-presentation competence	Tumour biopsy	HLA-I, B2M, TAP1/2, antigen-processing genes; MHC-I/II by IHC, IF, or flow cytometry	Preserved HLA-I/B2M/TAP1/2 and antigen-processing machinery	Loss or downregulation of HLA-I, B2M, TAP1/2, or antigen-processing genes
Dendritic-cell licensing and nodal priming	Tumour biopsy; TDLN aspirate when feasible	cDC1 markers: XCR1, CLEC9A, BATF3, CD141; activation/migration markers: CD40, CD80, CD86, CCR7, HLA-DR	Enriched cDC1 and activated/migratory DC signatures compatible with cross-presentation and lymph-node priming	Scarce cDC1 signatures; poor DC activation; weak CCR7-dependent migratory profile
Intratumoural T-cell substrate	Tumour biopsy; invasive margin	CD8^+^ T cells; spatial immune profiling; GZMB, PRF1, IFNG; TCF1^+^PD-1^+^ progenitor-like T cells when feasible	CD8^+^ infiltration with cytotoxic or progenitor-like features and tumour proximity	Sparse CD8^+^ T cells, immune exclusion, absent cytotoxic genes, or terminal dysfunction without priming
Peripheral immune activation	Peripheral blood	Flow cytometry or CyTOF for Ki-67^+^ CD8^+^ T cells, activated CD8^+^ T cells, myeloid activation markers; IFN-stimulated gene induction	Post-vaccine or post-conditioning increase in activated/proliferating CD8^+^ T cells, transient IFN-linked activation, or myeloid activation	No detectable systemic immune activation after vaccination or immune conditioning
T-cell clonal dynamics	Peripheral blood; tumour biopsy; paired longitudinal samples	TCR sequencing; antigen-specific T-cell assays; longitudinal repertoire tracking	TCR clonal expansion, repertoire broadening, vaccine-reactive clones, or epitope spreading	No clonal expansion, stagnant repertoire, or failure to expand vaccine/tumour-reactive clones
Composite immune-readiness interpretation	Integrated tumour, blood, and optional TDLN assessment	Multimodal integration of transcriptomic, cellular, spatial, and clonal readouts	Multiple upstream modules converge: innate activation, antigen presentation, DC licensing, T-cell priming, trafficking, and effector substrate	Coordinated absence of these modules despite preserved tumour antigenicity or checkpoint expression

The practical implication is that ICIs should be understood as interventions acting on a system, a coupled host–tumor immunological state, rather than as drugs directed at a tumor cell feature (25). This is already implicit in the foundational checkpoint blockade paradigm: durable responses occur most consistently when pre-existing antitumor T cells exist and are restrained by inhibitory pathways, whereas resistance frequently reflects upstream failures in innate activation, antigen presentation, or the quality and deployability of effector responses (30). Framed this way, immune readiness becomes the central variable that reconciles biomarker observations (T cell–inflamed and IFN-responsive states) with mechanism (innate sensing, cross-presentation, clonal expansion, and inflammatory plasticity): checkpoint blockade does not primarily act on tumors in isolation; it acts on the readiness of the host immune system to execute a coordinated antitumor response (3, 6).

## Vaccines as platforms for immune priming rather than direct tumor control

Therapeutic cancer vaccination has repeatedly demonstrated a paradox: it can be highly immunogenic and yet clinically weak as a stand-alone intervention in advanced disease. This is not a contradiction so much as a misalignment between what most cancer vaccines have historically been optimized to do (deliver antigen and elicit measurable circulating T cell responses) and what established tumors require for regression (a coordinated, tissue-effective immune programme that begins with competent innate activation, proceeds through properly licensed DC priming, and culminates in sustained effector function at the tumor site) (35, 36). Classic reviews already framed this gap: preventive vaccines are exceptionally successful because they block infection via durable humoral immunity, whereas therapeutic vaccines against established malignancy must induce robust cell-mediated immunity and therefore depend on tight collaboration between innate APCs (especially activated DCs) and adaptive CD4^+^ and CD8^+^ T cell programs (37, 38). In that sense, the most realistic and clinically productive reframing is not that vaccines kill tumors directly, but that they can program the immune system into a state in which other therapies, most notably checkpoint blockade, acquire real biological leverage (7, 39).

This reframing becomes intuitive once one recognizes what immune checkpoints *do not* do. PD-1/PD-L1 blockade cannot manufacture tumor-reactive T cells from nothing; it amplifies and restores function primarily when antigen-experienced T cells exist and when the tumor milieu is permissive to trafficking, restimulation, and execution (7, 35, 40). Therefore, the bottleneck in many cold tumors is upstream: insufficient innate ignition, inadequate DC cross-priming, and poor recruitment of effectors into malignant tissue (26, 39). Tumor-intrinsic immune evasion programs can actively enforce this upstream failure by suppressing priming and T cell infiltration, limiting the substrate on which checkpoint inhibition can act (20, 41). In that setting, the most valuable contribution of a vaccine is not direct cytotoxicity, but the ability to supply (i) antigenic instruction, (ii) inflammatory context, and (iii) DC licensing signals that collectively initiate a productive cancer–immunity cycle (37, 38).

A key anatomical site where this vaccine-enabled immune readiness is initiated is the tumor-draining lymph node (TDLN). TDLNs are not passive conduits for tumor antigens, but organized immune hubs in which migratory DCs transport tumor-derived or vaccine-delivered antigens, acquire maturation signals, and prime naïve or stem-like tumor-reactive T cells (34, 42). Within this nodal compartment, vaccination can enhance antigen presentation, co-stimulatory signalling, type I interferon–linked DC licensing, and clonal expansion of CD8^+^ T cells before these cells enter the circulation and infiltrate tumor tissue. This nodal priming step is particularly relevant to checkpoint blockade because TDLNs can preserve reservoirs of stem-like or progenitor-like tumor-specific CD8^+^ T cells that sustain ongoing antitumor immunity and provide the cellular substrate for PD-1/PD-L1 blockade (34, 43). Thus, vaccines do not simply increase immune activity at the tumor site; they can mobilize a systemic priming circuit in which TDLN activation precedes and supports intratumoral immune conversion. In this view, effective vaccination-enabled checkpoint therapy requires alignment between antigen delivery, adjuvant-driven innate activation, TDLN priming, and subsequent effector deployment into the tumor microenvironment.

Mechanistically, therapeutic vaccination is best understood as a platform that couples antigen delivery to innate pattern recognition and DC maturation. Reviews of therapeutic vaccine designs emphasize that, regardless of whether the platform is peptides, proteins, nucleic acids, viral vectors, or DC vaccines, successful therapeutic vaccination requires antigen presentation by MHC molecules on appropriately activated DCs, because this is essential for the induction of robust therapeutic T cell responses (37–39, 44, 45). DC-based vaccine frameworks similarly highlight that effective therapeutic vaccination must both prime naïve T cells and remodel dysfunctional pre-existing T cell states (including PD-1^+^, anergic, or chronically stimulated populations), with the explicit goal of generating long-lived memory CD8^+^ T cells that can prevent relapse rather than producing only transient effectors (46–48).

The ability of therapeutic vaccines to engineer immune readiness depends not only on antigen delivery, but also on adjuvant-driven innate immune programming (35, 49). Adjuvants engage pattern-recognition and inflammatory sensing pathways, including Toll-like receptors, NOD-like receptors, inflammasome-associated sensors, STING-related circuits, and innate pathways activated by saponins, emulsions, nanoparticles, or viral vectors. These signals promote dendritic-cell maturation, MHC and co-stimulatory molecule expression, cytokine release, lymph-node migration, and cross-presentation, thereby converting antigen exposure into productive T-cell priming rather than tolerance (50). Distinct adjuvant classes imprint different immune states: TLR3, TLR7/8, and TLR9 agonists favour type I interferon–dominated programs and cDC1 licensing; TLR4 agonists and bacterial-derived adjuvants promote inflammatory myeloid activation; inflammasome-linked adjuvants enhance IL-1β/IL-18-driven inflammation; and saponin or nanoparticle systems improve antigen uptake and lymph-node delivery (35, 51). In the context of checkpoint blockade, these adjuvant-induced innate programs create the immune substrate required for PD-1/PD-L1 or CTLA-4 inhibition to act productively. Thus, adjuvant selection, dose, route, and timing should be treated as central design variables in vaccine–checkpoint combinations (49, 51).

This focus on quality (durability, functionality, tissue access) over quantity (circulating frequency) is reinforced by immunoguiding concepts: many trials have shown immunogenicity and safety, yet overall clinical efficacy has been low, and simple measures of circulating effector responses are often insufficient predictors of outcome; what matters is whether effector cells can migrate to, persist within, and function in the TME (26). In other words, a vaccine’s principal clinical job in established disease is to build the immune engine (priming, licensing, trafficking, persistence), not to serve as the entire vehicle of tumor eradication (26, 36).

Critically, the historical underperformance of many vaccine monotherapies has not only been due to tumor-mediated suppression; it has also reflected avoidable design features that misdirect vaccine-primed T cells away from tumors (36, 52). A particularly instructive mechanistic example is persistent antigen depots created by incomplete Freund’s adjuvant (IFA), widely used in peptide vaccine trials. In preclinical systems, peptide/IFA vaccination could prime tumor-specific CD8^+^ T cells, but those primed cells preferentially accumulated at the antigen-rich vaccination site rather than at tumors, where they became dysfunctional and underwent deletion (52, 53). Even adding additional immune stimulants (CD40 agonism, TLR7 agonism, IL-2) reduced apoptosis but did not eliminate sequestration, whereas shifting to a non-persisting formulation redirected T cell localization toward tumors and improved antitumor activity (52). The broader implication is foundational for vaccination-enabled logic: antigen delivery is necessary, but not sufficient, spatiotemporal control of antigen persistence and inflammatory context determines whether vaccine-elicited T cells become tumor-seeking effectors or are trapped and exhausted in non-productive niches (52, 54). This helps explain why vaccines can look immunogenic on paper (or in blood) yet fail clinically, and it also points to why modern platforms that better coordinate antigen kinetics and innate activation can function as superior priming tools (36).

Modern neoantigen vaccine trials provide concrete proof-of-principle that vaccines can drive robust priming, clonal expansion, and functional tumor targeting, yet still underscore why monotherapy is usually not the right expectation. Personalized neoantigen vaccines in melanoma have been shown to induce polyfunctional CD4^+^ and CD8^+^ T cell responses against multiple neoepitopes, with evidence of tumor infiltration and epitope spreading, indicating that vaccines can indeed generate the repertoire breadth and functional quality required for tumor control (55–57). At the same time, these studies also illustrate that the clinical path to maximal benefit often involves either subsequent checkpoint blockade or combination strategies that maintain effector function and overcome adaptive resistance, consistent with the concept that vaccines can light the fire, while checkpoint blockade helps prevent extinguishing once tumors respond by upregulating inhibitory pathways (3, 39, 55, 56, 58, 59).

The key step linking these observations is the recognition that vaccines are not merely antigen carriers but immune-programming devices (35). Vaccine adjuvants and delivery systems shape the innate cues that license DCs and instruct the quality of downstream T cell responses; the field increasingly treats adjuvant-driven innate programming as a central determinant of whether therapeutic vaccination yields productive tumor immunity. In parallel, T cell biology reminds us that durable tumor control requires not only expansion but the right differentiation trajectory—memory-competent, proliferative, and persistent populations outperform short-lived terminal effectors in sustaining antitumor immunity (56, 60). These principles collectively motivate the platform view: vaccines are best positioned to create or reshape the upstream immune state, innate activation, DC licensing, repertoire priming, and memory-biased expansion, upon which ICIs can then act effectively (61).

Finally, the monotherapy-versus-enabler distinction becomes clearer when viewed through the lens of what therapeutic vaccination must overcome in patients with established cancer. Editorial perspectives on vaccine development have long emphasized that, despite abundant evidence of immunogenicity in hundreds of trials, clinical efficacy has generally been low, partly because tumors develop slowly under immune pressure and can induce tolerance, mispolarization, and suppressive responses that vaccines may inadvertently expand if not designed and monitored carefully (36). This is precisely why vaccines should be positioned as enablers, tools to purposefully program immune conditions (and monitor them) so that downstream interventions, especially checkpoint blockade, can convert newly primed or reinvigorated T cells into durable tumor control, rather than asking vaccination alone to overcome every barrier simultaneously (38, 62).

Taken together, a more precise and credible paradigm is that therapeutic cancer vaccines are rarely directly cytotoxic on their own, but they can initiate and shape the immune circuits that make tumor killing feasible: systemic innate activation that licenses antigen presentation, competent DC-driven priming that expands tumor-specific CD8^+^ clones, and differentiation programs that support trafficking, persistence and function in tissues (37, 38). Historical limitations, including non-physiologic antigen depots that sequester and delete primed T cells, explain why immunogenicity has not reliably translated into clinical benefit, and they directly inform how next-generation platforms should be engineered (52, 63). Modern neoantigen vaccination demonstrates that potent priming and repertoire broadening are achievable in humans, reinforcing the idea that the most rational clinical role of vaccines in advanced cancer is to *build immune readiness*, and thereby convert checkpoint blockade from an inert brake-release into an effective amplifier of a newly established antitumor response (56).

## Type I interferon–driven innate reprogramming as a prerequisite for tumor sensitization

A recurring insight emerging from both mechanistic studies and clinical observations is that immune checkpoint blockade becomes effective only once a tumor-bearing host has undergone a prior phase of innate immune reprogramming (64, 65). Central to this transition is IFN-I, which functions not as a generic inflammatory signal but as a systems-level coordinator that links tumor sensing to myeloid activation, DC licensing, and adaptive immune engagement. In non-inflamed tumors, the dominant limitation is frequently not the absence of checkpoint targets, but the absence of this IFN-I–dependent instructional layer that enables antigen presentation, effector recruitment, and productive T cell priming (27, 64–67).

At the level of innate immunity, IFN-I acts as a primary axis of myeloid activation. Tumor-derived danger signals sensed through innate pathways converge on IFN-I production, which in turn induces maturation, migration, and functional competence of APCs. This IFN-I–conditioned myeloid state is required for effective cross-presentation of tumor antigens and for the establishment of chemokine gradients that permit immune cell trafficking (65). Without this step, tumors remain immunologically silent despite the presence of potential antigens, and downstream adaptive immunity fails to initiate in a coordinated manner (20, 67).

Functionally, IFN-I provides the critical bridge between innate and adaptive immunity. Conventional DCs, particularly cross-presenting cDC1 populations, depend on IFN-I signalling to upregulate antigen-processing machinery, co-stimulatory molecules, and lymph node migratory programs (27, 66). In parallel, IFN-I shapes the activity of antigen-presenting macrophages, promoting a transient inflammatory phenotype capable of supporting T cell priming rather than enforcing tolerance or exclusion. Together, these IFN-I–licensed myeloid compartments enable effective lymph node priming and expansion of tumor-reactive CD8^+^ T cell clones, a prerequisite for any subsequent therapeutic reinvigoration (22).

This IFN-I–driven priming phase also explains why PD-L1 expression frequently emerges *after* immune activation rather than preceding it (27). PD-L1 upregulation is often an inducible, physiological consequence of IFN-stimulated immune engagement, reflecting adaptive counter-regulation in response to active T cell pressure (66–69). In this context, checkpoint pathways become relevant only once IFN-I–dependent priming has occurred; PD-1/PD-L1 blockade then acts to prevent premature functional attenuation of newly activated effector cells. Thus, inducible PD-L1 is not a marker of checkpoint dominance *per se*, but an indicator that the immune system has entered a state in which checkpoint inhibition can exert meaningful effects (70, 71).

Crucially, IFN-I signalling is not monolithic, and its biological consequences depend on timing, magnitude, and cellular context (68, 69). Physiological, spatially restricted IFN-I signalling supports DC licensing, effector recruitment, and adaptive priming. By contrast, sustained or dysregulated IFN-I exposure can drive chronic interferon-stimulated gene programs associated with immune exhaustion, compensatory inhibitory ligand expression, and resistance to immunotherapy (68, 69). Clinical and transcriptional analyses identify interferon-responsive resistance states in which persistent IFN signalling coexists with diminished responsiveness to ICIs, underscoring that IFN-I must be programmed, not merely amplified.

Tumor-intrinsic factors can further modulate whether IFN-I–dependent reprogramming occurs at all. Oncogenic pathways such as Wnt–β-catenin signalling can suppress chemokine production required for DC recruitment, effectively insulating the tumor from innate immune sensing and preventing IFN-I–mediated priming (20, 22). In such settings, tumors appear cold not because they lack immunogenicity, but because they fail to enter an IFN-instructed immune trajectory upstream of checkpoint biology (20).

Collectively, these observations support a unified model in which IFN-I–driven innate reprogramming constitutes the decisive step in tumor sensitization. The emergence of a hot tumor phenotype reflects an induced immunological state characterized by myeloid activation, licensed antigen presentation, effective lymph node priming, and coordinated effector deployment, coupled with adaptive regulatory feedback that renders checkpoint pathways biologically relevant. Importantly, this state is neither fixed nor tumor-intrinsic; it is dynamically shaped by host innate signalling and can be modulated therapeutically (27, 72).

## Temporal immuno-engineering: sequence and timing as critical therapeutic variables

Because checkpoint blockade acts on ongoing T cell responses rather than initiating them, combination immunotherapy is inherently sequential: an upstream priming and activation phase must first generate antigen-experienced effector and progenitor pools, and only then can PD-1/PD-L1 blockade productively release inhibitory constraints to amplify function, broaden epitope breadth, and sustain tumor control (73–75) (**Figure 1**). This logic is grounded in T cell biology rather than therapeutic preference, as PD-1 is rapidly induced upon TCR engagement and PD-L1 expression is frequently inducible in response to inflammatory cues. Consequently, both the cellular substrate targeted by checkpoint inhibitors and the ligand landscape they engage are dynamic rather than static, rendering timing a mechanistic variable rather than a logistical detail (76, 77).

Importantly, this temporal logic should not be interpreted as a rigid clinical requirement that vaccination must always precede checkpoint blockade. Rather, the central principle is biological alignment: innate activation, antigen presentation, T cell priming, and checkpoint release must converge within a therapeutic window in which vaccine-induced or vaccine-amplified immune responses can be productively sustained by PD-1/PD-L1 blockade (77–79). Accordingly, vaccination may be integrated before, during, or in close temporal coordination with checkpoint inhibition, depending on the vaccine platform, tumour context, and treatment regimen. The goal is therefore not to impose a universal vaccine-before-ICI sequence, but to ensure that checkpoint blockade acts on an immune system that has been adequately primed, activated, or reconditioned.

Preclinical sequencing studies illustrate this principle with particular clarity. In tumor models resistant to anti–PD-1 therapy, simultaneous vaccination and PD-1 blockade can produce synergy, whereas administering PD-1 blockade before antigen priming abolishes the therapeutic benefit of the combination (77, 80). This defines a distinct failure mode that can be conceptualized as unproductive checkpoint release: inhibitory pathways are blocked in a system that has not yet generated sufficient antigen-driven activation to convert checkpoint release into productive effector expansion (81, 82). Importantly, this loss of efficacy is accompanied by a failure to accumulate vaccine-induced antigen-specific CD8^+^ T cells within the TME, demonstrating that sequence directly governs clonal deployment rather than merely the magnitude of response (76, 80). Mechanistically, premature PD-1 blockade under suboptimal priming conditions can promote dysfunctional PD-1^+^CD38^hi^ CD8^+^ T cell states associated with resistance, whereas appropriately timed priming reverses this phenotype and restores responsiveness (83).

Independent experimental work converges on the same conclusion while refining the temporal window. In vaccination models, PD-1 blockade delivered during the period of CD8^+^ T cell activation produces superior antitumor responses compared with administration before or after priming. Disruption of the PD-1–PD-L1 axis at the time of T cell activation yields CD8^+^ T cells with enhanced effector differentiation while preserving a smaller progenitor-like subset, translating into improved tumor control upon transfer (75, 77, 83, 84). These data indicate that a substantial component of checkpoint blockade efficacy in vaccine combinations arises from its effects on fate decisions made during priming, rather than exclusively from reinvigoration of terminally exhausted T cells in established tumors (77, 80). In this light, administering vaccination before immune checkpoint inhibition, or combining vaccination with checkpoint blockade during the priming phase, is mechanistically aligned with PD-1 biology. By contrast, delivering checkpoint inhibition prior to vaccination risks acting on an immune system that lacks the appropriate cellular substrate or has already entered a dysfunctional trajectory (39, 73, 74).

This sequencing logic naturally unfolds over days to weeks in clinically realistic regimens (85). In a first-line non-small-cell lung cancer study combining a personalized neoantigen vaccine with chemotherapy and pembrolizumab, patients underwent an initial systemic therapy phase followed by a structured vaccination schedule with priming and booster doses administered while PD-1 blockade continued (84). This design illustrates temporal immuno-engineering in practice: an initial preparatory interval, a priming phase characterized by clonal expansion and diversification, and a maintenance phase during which checkpoint blockade sustains effector function. Notably, epitope spreading to non-vaccinated neoantigens was detected after vaccination, directly linking post-priming timing to repertoire broadening (85).

At the level of T cell clonal ecology, epitope spreading is itself sequence-dependent (81, 86). It requires an initial wave of priming against dominant antigens, subsequent tumor cell killing and antigen release, and secondary priming events that rescue subdominant clones from immunodominance and suppressive dynamics (86). Experimental models demonstrate that PD-1 blockade enhances the persistence and expansion of subdominant CD8^+^ T cell clones, thereby facilitating epitope spreading when delivered after priming has established a competitive clonal hierarchy (86, 87). This provides a mechanistic rationale for why the benefits of checkpoint blockade on repertoire breadth emerge most clearly after the priming phase and over extended temporal windows.

Clinical translational studies further reinforce that sequence matters because priming actively remodels the TME (82, 85). In melanoma patients treated with a multi-peptide vaccine combined with nivolumab, on-treatment biopsies revealed increased PD-L1 expression, upregulation of antigen-presentation machinery, and enhanced immune infiltration relative to baseline (82). These findings underscore a critical temporal point: PD-L1 expression and antigen presentation competence are often induced consequences of immune activation rather than fixed baseline features (81, 84, 88). Administering checkpoint blockade before these programs are established risks releasing inhibitory constraints in a system that has not yet built the track on which T cells can operate.

Broader conceptual syntheses of combination immunotherapy emphasize that synergy with ICIs frequently requires upstream conversion of non-inflamed tumors into inflamed states, rather than assuming that concurrent administration automatically overcomes resistance (12, 16, 22). Within this framework, unproductive checkpoint release should be understood not as a universal contraindication to early ICI use, but as a context-dependent failure mode that arises when blockade is temporally uncoupled from effective priming—either because priming is absent, delayed, or structurally impaired (84).

Finally, timing is clinically relevant at the level of the host immune system as a whole (88). Large observational analyses in ICI-treated patients indicate that systemic immune activation events, including vaccination, can intersect with checkpoint therapy on clinically meaningful timescales, influencing survival outcomes even when the vaccine is not tumor-specific (88–90). Although such observations do not establish causality, they reinforce the principle that the host immune state is dynamic and that exogenous immune perturbations can shift the baseline upon which ICIs act.

Taken together, these data support a coherent temporal model of immunotherapy: effective vaccination establishes and programs antigen-experienced CD8^+^ T cell populations, remodels antigen presentation and inflammatory tone, and creates the conditions under which PD-1/PD-L1 blockade can amplify function, preserve critical clones, and enable epitope spreading (88). Conversely, administering checkpoint blockade before or outside the priming window can yield diminished benefit and, under suboptimal conditions, may actively induce dysfunctional immune states consistent with resistance—an outcome best described as unproductive checkpoint release. The practical implication is clear: sequence is not a technical footnote; it is part of the mechanism (77, 91, 92).

## Clinical evidence supporting vaccine-mediated sensitization to checkpoint inhibition

Clinical evidence supporting tumor-directed antigen vaccines combined with checkpoint blockade converges on a coherent mechanistic principle: immune checkpoint inhibition cannot generate antitumor immunity *de novo* but instead amplifies, stabilizes, and functionally sustains tumor-specific T cell responses that must first be induced through effective antigenic priming. Tumor-directed vaccines address this upstream bottleneck by increasing the effective antigenic load perceived by the immune system, expanding the breadth and magnitude of tumor-specific T cell repertoires, and promoting productive priming within secondary lymphoid tissues. Within this framework, checkpoint blockade functions as a downstream enabler rather than a primary initiator of immunity, converting vaccine-induced T cell populations into durable effector and memory responses capable of tumor control (39) (**Figure 1**).

The most compelling clinical validation of this model comes from personalised neoantigen vaccination strategies combined with PD-1 blockade. In resected high-risk melanoma, the randomised phase IIb KEYNOTE-942 trial evaluating the individualised mRNA neoantigen vaccine mRNA-4157 (V940) in combination with pembrolizumab demonstrated a significant reduction in the risk of recurrence or death compared with pembrolizumab alone (NCT03897881) (59). This study provided the first controlled evidence that tumor-directed vaccination can meaningfully sensitize disease to checkpoint inhibition in the adjuvant setting. Immunological analyses revealed robust induction and expansion of neoantigen-specific CD8^+^ and CD4^+^ T cell clonotypes, accompanied by epitope spreading beyond the encoded targets, indicating that vaccination broadened tumor antigen recognition rather than merely amplifying pre-existing immunity (59).

Mechanistic support for vaccine-mediated sensitisation in immunologically refractory tumors is provided by studies in pancreatic ductal adenocarcinoma. In a phase I trial of resected pancreatic cancer, administration of a personalised mRNA neoantigen vaccine (autogene cevumeran) combined with PD-L1 blockade (atezolizumab) and chemotherapy induced *de novo* neoantigen-specific T cell responses in approximately half of treated patients, with immune responders experiencing prolonged recurrence-free survival compared with non-responders (NCT04161755) (93). Longitudinal T cell receptor sequencing demonstrated sustained clonal expansion and recall of vaccine-induced T cells, establishing that vaccination reshaped the tumor-specific repertoire and created immune substrates upon which checkpoint blockade could act (93).

Evidence from advanced disease settings further supports the generalisability of this paradigm. In hepatocellular carcinoma, a personalised DNA neoantigen vaccine (GNOS-PV02) co-administered with plasmid IL-12 and pembrolizumab produced objective responses, including complete remissions, alongside clear induction of neoantigen-specific T cell immunity and intratumoral immune activation (NCT04251117) (94). Clinical benefit correlated with the number of encoded neoantigens and the magnitude of vaccine-induced T cell expansion, directly linking antigenic breadth to checkpoint sensitisation (94).

Tumor-associated antigen vaccines targeting shared self-antigens provide additional, albeit more nuanced, support for this model. In melanoma, the telomerase-derived peptide vaccine UV1 combined with pembrolizumab demonstrated durable clinical responses accompanied by vaccine-specific T cell immunity and favourable immune remodelling of the TME (NCT03538314) (95). Similarly, the RNA-based FixVac platform (BNT111), encoding melanoma-associated antigens, induced potent systemic and intratumoral T cell responses and showed clinical activity when administered with PD-1 blockade, including in patients previously exposed to checkpoint inhibitors (NCT02410733) (96). Together, these studies indicate that sufficiently immunogenic antigen-delivery platforms can partially overcome tolerance barriers associated with shared tumor antigens when combined with checkpoint inhibition.

Viral vector–based tumor vaccines further illustrate the dependence of checkpoint efficacy on antigen-specific priming. In patients with incurable HPV16-positive cancers, long-peptide vaccination targeting E6 and E7 antigens combined with nivolumab resulted in objective responses and survival outcomes exceeding historical expectations for PD-1 monotherapy (NCT02426892) (97). Treatment efficacy correlated with expansion of HPV-specific T cell populations, providing a particularly clear demonstration that antigen-specific priming is a prerequisite for effective checkpoint-mediated tumor control.

Conversely, negative or modest trials are equally instructive in defining the limits of tumor-directed vaccination. A phase I study combining a shared neoantigen vaccine with nivolumab and ipilimumab failed to produce objective responses despite acceptable safety (NCT01970358), largely owing to immunodominance effects that skewed T cell responses towards non-protective epitopes (57). Earlier studies using whole-cell vaccines such as GVAX combined with CTLA-4 blockade (ipilimumab) in pancreatic cancer produced limited clinical benefit but demonstrated expansion of tumor-specific T cell responses in patients with prolonged survival (NCT00836407), reinforcing the principle that antigenic priming can alter immune trajectories even when efficacy remains modest (98). Similarly, vaccination with a p53-encoding viral vector combined with pembrolizumab resulted in disease stabilisation preferentially among patients mounting robust p53-specific T cell responses, again linking antigen-directed priming to checkpoint responsiveness (NCT02432963) (99).

Collectively, these data establish that tumor-directed antigen vaccines sensitise cancers to checkpoint inhibition by actively constructing the tumor-specific T cell repertoire required for checkpoint blockade to exert therapeutic effects (100). Rather than functioning as adjuncts, these vaccines operate upstream of checkpoint pathways, transforming antigen-poor or clonally restricted immune landscapes into systems capable of responding to inhibitory checkpoint release. Across tumor types, antigen platforms, and clinical settings, a consistent pattern emerges in which checkpoint inhibitors amplify immunity that vaccines must create, positioning tumor-directed vaccination as a mechanistically grounded strategy to overcome primary resistance to checkpoint blockade (39, 88).

Whereas tumor-directed vaccines construct the antigenic substrate required for checkpoint efficacy, a parallel body of clinical evidence indicates that vaccines can also sensitise tumors by reprogramming the immune state of the host itself (101). Clinical evidence supporting immune-modulatory and non-tumor antigen vaccines combined with checkpoint blockade, therefore, defines a distinct but complementary sensitisation paradigm in which vaccines do not primarily expand tumor-specific T cell repertoires but instead reconfigure basal immune readiness. In this model, vaccination resets innate activation thresholds, licenses APCs, induces IFN-I–dominated inflammatory programmes, and reshapes systemic immune tone, enabling checkpoint blockade to sustain and amplify antitumor immunity that emerges as a consequence of global immune reconditioning rather than direct tumor antigen targeting (14, 39, 88).

One of the most clinically mature examples of immune-modulatory vaccination is sipuleucel-T, an autologous cellular vaccine that induces systemic immune activation while broadly enhancing antigen presentation. In metastatic castration-resistant prostate cancer, administration of sipuleucel-T followed by CTLA-4 blockade demonstrated that vaccination induces immune activation accompanied by compensatory checkpoint upregulation, thereby creating a biologically permissive setting for checkpoint inhibition (102). In a phase II study evaluating sipuleucel-T with ipilimumab (NCT01804465), clinical activity was modest, but immune analyses revealed pronounced CD4^+^ and CD8^+^ T cell activation, with baseline immune state strongly predicting clinical benefit. Patients with lower frequencies of CTLA-4–expressing T cells before therapy were more likely to respond, underscoring that host immune tone conditions responsiveness to checkpoint blockade following immune-modulatory vaccination (102).

The importance of sequencing and systemic immune context was further highlighted in a phase Ib trial testing different administration orders of sipuleucel-T and PD-L1 blockade with atezolizumab in metastatic prostate cancer (NCT03024216) (103). Although objective responses were rare, vaccination increased T cell receptor diversity and promoted immune activation irrespective of sequence, reinforcing that immune-modulatory vaccines act primarily by reshaping global immune readiness rather than directly inducing tumor-specific cytotoxicity.

Live-attenuated and microbial vaccines provide additional evidence that non-tumor antigens can reprogramme immunity in a manner that sensitises tumors to checkpoint blockade. Intralesional administration of *Bacillus Calmette–Guérin* (BCG), long known to induce potent innate and adaptive immune activation, has been evaluated in combination with CTLA-4 and PD-1 inhibitors. In advanced melanoma, intralesional BCG followed by ipilimumab resulted in excessive immune activation and unacceptable toxicity without clinical benefit (NCT01838200), illustrating that uncontrolled innate stimulation can overshoot therapeutic windows and emphasising the need for calibrated immune modulation rather than maximal inflammation (104). By contrast, in non-muscle-invasive bladder cancer, intravesical BCG combined with systemic pembrolizumab was well tolerated and associated with high rates of disease control in patients refractory to prior BCG therapy (NCT02324582), providing clinical evidence that anatomically constrained innate activation can synergise with checkpoint blockade when appropriately dosed (105).

Oncolytic viruses represent a related class of immune-modulatory agents that sensitise tumors to checkpoint inhibition by inducing local inflammation, IFN-I signalling, and antigen presentation without encoding tumor-specific antigens. In advanced melanoma, the intratumoral administration of the oncolytic Coxsackievirus A21 (V937) in combination with pembrolizumab resulted in high objective response rates, including durable complete responses, even in tumors lacking baseline immune infiltration (NCT02565992) (106). Responses were associated with systemic cytokine induction and chemokine gradients consistent with innate immune activation rather than pre-existing adaptive immunity, indicating that viral vaccination can reset immune tone and convert immunologically cold tumors into checkpoint-responsive lesions.

Targeting immunoregulatory pathways through vaccination further illustrates how non-tumor antigen strategies can condition responsiveness to checkpoint blockade. Indoleamine 2,3-dioxygenase (IDO), a key mediator of immune tolerance and resistance to CTLA-4 blockade, has been targeted using long-peptide vaccination in metastatic melanoma. In a phase I study combining an IDO-derived peptide vaccine with ipilimumab, treatment was generally safe and induced vaccine-specific T cell responses in a subset of patients, with signals of disease stabilisation exceeding historical expectations for ipilimumab alone (NCT02077114) (107). Although efficacy was limited, these results demonstrate that vaccination against immunoregulatory enzymes can partially reverse immune suppression and reshape the host immune landscape in a manner complementary to checkpoint inhibition.

The most conceptually transformative evidence for immune-modulatory vaccination comes from recent studies demonstrating that clinically approved non-oncologic mRNA vaccines can sensitise tumors to checkpoint blockade. SARS-CoV-2 mRNA vaccines induce a systemic, viraemia-like innate immune response characterised by robust IFN-I production, myeloid activation, and enhanced antigen presentation, thereby resetting the cancer–immunity cycle. Retrospective analyses across large cohorts of patients with non-small-cell lung cancer and metastatic melanoma revealed that receipt of a COVID-19 mRNA vaccine within 100 days of initiating immune checkpoint therapy was associated with significantly improved overall survival, including in immunologically cold tumors, without evidence of increased immune-related toxicity (88). These clinical observations were supported by mechanistic data demonstrating increased tumor PD-L1 expression, APC licensing, and epitope spreading following vaccination, establishing non-tumor antigen mRNA vaccines as potent systemic immune modulators capable of conditioning checkpoint responsiveness.

Collectively, these clinical data redefine checkpoint blockade as a context-dependent therapy whose efficacy can be engineered upstream by vaccines that shape antigen availability or, more fundamentally, the host’s immune state. By acting on innate activation, inflammatory tone, and APC function, immune-modulatory vaccines convert checkpoint inhibition from a narrow intervention into a strategy capable of operating across a broad spectrum of immune landscapes, thereby expanding the therapeutic reach of immunotherapy beyond antigen-rich tumors.

## Tumor-agnostic vaccination strategies as systemic innate immune modulators

Clinical evidence increasingly supports a tumor-agnostic vaccination paradigm in which the principal barrier to immune checkpoint efficacy is not tumor antigen availability, but the failure of systemic innate immune activation (108, 109). In these contexts, immune checkpoint blockade is constrained by an unresponsive or improperly conditioned innate immune compartment, rather than by insufficient tumor antigenicity. Tumor-agnostic vaccines, particularly viral or microbe-derived platforms, can overcome this bottleneck by acting as systemic innate immune modulators, reprogramming host immune readiness upstream of antigen-specific adaptive responses (5, 110).

A central mechanistic foundation for this paradigm is the concept of trained immunity, whereby innate immune cells acquire durable functional reprogramming following vaccination or infection (111, 112). Live attenuated vaccines such as BCG induce long-lasting epigenetic and metabolic rewiring of monocytes and myeloid progenitors, leading to enhanced cytokine production, increased antigen-presenting capacity, and heightened responsiveness to secondary immune challenges independent of antigen specificity (111, 113, 114). This innate memory state lowers activation thresholds for subsequent immune stimulation, providing a permissive systemic context in which checkpoint blockade can effectively amplify antitumor immunity (109, 111).

Clinical observations suggest that such innate reprogramming can directly influence responsiveness to ICIs. Chronic interferon signalling and innate immune activation shape tumor–immune interactions by regulating DC licensing, chemokine gradients, and T cell trafficking, all of which are prerequisites for effective checkpoint-mediated tumor control (20, 68). Importantly, tumor-agnostic vaccination does not supply tumor antigens *de novo* but instead restores the upstream innate signals required for endogenous antigen presentation and T cell recruitment to occur (5, 20).

Oncolytic viruses exemplify this strategy in a clinically actionable form (108, 115). Viral vaccines and virotherapies function as potent, off-the-shelf innate immune activators that induce local and systemic IFN-I responses, myeloid cell recruitment, and intratumoral inflammation without reliance on tumor-specific antigen encoding (110, 115). Clinical trials combining oncolytic viruses with PD-1 blockade in melanoma demonstrated high response rates, including complete remissions in patients whose tumors lacked baseline T cell infiltration, underscoring that innate immune activation can convert immunologically cold tumors into checkpoint-responsive lesions (115–118). These responses were associated with *de novo* CD8^+^ T cell infiltration, increased PD-L1 expression, and interferon-driven chemokine induction, consistent with immune awakening rather than antigen targeting.

Non-oncologic viral vaccines further reinforce the tumor-agnostic principle. Systemic viral vaccination induces a transient, infection-like immune state characterised by innate immune activation, checkpoint receptor upregulation, and broad immune mobilisation (119). Viral infections, including SARS-CoV-2, are associated with coordinated induction of inhibitory immune receptors and interferon-regulated pathways, revealing how viral immune activation naturally interfaces with checkpoint biology (119). Retrospective clinical analyses indicate that administration of mRNA viral vaccines proximal to immune checkpoint therapy correlates with improved survival outcomes across tumor types, suggesting that systemic innate immune activation can condition responsiveness to checkpoint inhibition even in the absence of tumor-directed antigen delivery (88, 120).

The translational appeal of tumor-agnostic vaccination lies in its scalability, speed, and antigen independence. Unlike personalised neoantigen vaccines, these platforms are immediately deployable, manufacturable at scale, and agnostic to TMB, HLA genotype or antigen discovery pipelines. From a regulatory perspective, repurposing licensed vaccines or clinically validated viral platforms may offer accelerated development pathways compared with bespoke antigen-specific vaccines (5, 121).

However, the limits of this approach are equally well defined. Tumor-agnostic vaccination alone is insufficient to mediate durable tumor control and requires concomitant checkpoint blockade to prevent rapid immune exhaustion and adaptive resistance (108). Moreover, not all tumors respond, particularly those with profound immune exclusion, defective interferon signalling, or dominant suppressive myeloid programs (20, 109). Excessive or poorly calibrated innate activation also carries the risk of uncoupled inflammation, immune-related toxicity, and paradoxical resistance driven by chronic interferon signalling (110, 112). These risks are especially relevant for broad-spectrum immune-conditioning strategies because tumor-agnostic vaccines activate innate immune pathways without requiring predefined tumor-antigen specificity. As a result, poorly controlled systemic activation may induce cytokine-driven toxicity, excessive type I interferon signalling, bystander tissue inflammation, or amplification of immune-related adverse events when combined with checkpoint blockade (68, 122). Although these platforms do not classically produce antigen-specific on-target/off-tumor toxicity in the same manner as TCR- or CAR-based therapies, they may still intensify immune recognition of shared self-antigens, inflamed healthy tissues, or tissue-resident antigen-presenting niches (123). Thus, tumor-agnostic vaccination should not be interpreted as indiscriminate immune stimulation, but as calibrated immune conditioning that requires careful control of dose, route, timing, inflammatory amplitude, tissue localization, and patient selection (122). These constraints underscore that tumor-agnostic vaccination is not a universal substitute for antigen-directed strategies, but a complementary modality whose efficacy depends on immune context and therapeutic sequencing (68).

Taken together, these data establish tumor-agnostic vaccination as a disruptive but mechanistically grounded strategy in cancer immunotherapy. In selected contexts, it is not necessary to direct the immune response toward specific tumor antigens; awakening the innate immune system may be sufficient to unlock the therapeutic potential of checkpoint blockade (5, 115). This reframes vaccination not solely as an antigen delivery platform, but as a systemic immune conditioning tool capable of reshaping the host–tumor immune interface upstream of adaptive specificity (111, 112). The translational challenge is therefore to define a therapeutic window in which systemic innate activation is strong enough to license antitumor immunity, but sufficiently constrained to avoid healthy-tissue inflammation, immune-related toxicity, and maladaptive chronic interferon signalling.

## Circadian and neuro-immune regulation of vaccine-induced antitumor immunity

Accumulating evidence indicates that vaccine-induced antitumor immunity does not arise in a static immunological landscape, but instead unfolds within temporally structured and neuro-regulated systems that critically shape immune priming, amplification, and checkpoint responsiveness (124, 125). Both innate and adaptive immune compartments are governed by intrinsic circadian clocks and extrinsic neural inputs that rhythmically regulate cytokine signalling, leukocyte trafficking, endothelial adhesion, and immune checkpoint expression (126, 127). As a consequence, the same immunological stimulus, whether vaccination or checkpoint blockade, can elicit qualitatively different outcomes depending on the time of administration and the prevailing systemic state (128). This temporal principle is consistent with emerging chrono-immunotherapy frameworks proposing that biological time should be treated as a design variable for advanced immunotherapies, including *in vivo* CAR T-cell platforms, where circadian regulation of leukocyte trafficking, vascular permissiveness, T-cell metabolism, exhaustion risk, and inflammatory toxicity may influence therapeutic performance (129).

At the cellular level, circadian clocks are embedded within key immune populations that mediate vaccine responses. CD8^+^ T cells display cell-intrinsic circadian oscillations driven by core clock components such as BMAL1 and CLOCK, which gate early activation, metabolic reprogramming, and proliferative capacity following antigen presentation (130–132). Experimental vaccination studies demonstrate that antigen-specific CD8^+^ T cell expansion, signalling through mTOR–AKT–IRF4 pathways, and subsequent effector differentiation are significantly enhanced when priming occurs during circadian phases permissive for T cell activation, whereas identical stimuli delivered at circadian troughs yield attenuated responses (130, 133). These findings establish that circadian timing modulates not only the magnitude of vaccine-induced immunity but also qualitative aspects of T cell programming that are directly relevant to durable antitumor responses.

DCs are similarly regulated by circadian clocks, which influence antigen uptake, migration to lymphoid tissues, and costimulatory molecule expression (134). Genetic disruption of circadian regulators in APCs abolishes time-of-day differences in T cell priming, underscoring that effective vaccination depends on coordinated circadian alignment across both innate and adaptive immune compartments (133, 135). Beyond immune cells, circadian oscillations in endothelial adhesion molecules and chemokine gradients regulate leukocyte trafficking into lymphoid organs and tumors, thereby shaping the spatial and temporal availability of immune effectors during vaccine-induced priming (126).

Within the TME, circadian control extends to immunosuppressive circuits that directly influence checkpoint efficacy. Myeloid-derived suppressor cells and PD-L1–expressing myeloid populations accumulate rhythmically, driven by clock-dependent cytokine secretion and epithelial–immune crosstalk (136, 137). Disruption of circadian regulation results in sustained immunosuppression, impaired cytotoxic T cell infiltration, and reduced responsiveness to immune checkpoint blockade, whereas time-of-day–optimised delivery of anti-PD-1 or anti-PD-L1 therapy restores antitumor activity in preclinical models (136).

Circadian oscillations also govern innate immune signalling pathways central to vaccine responsiveness. IFN-I production, pattern-recognition receptor signalling, and inflammatory cytokine release exhibit pronounced diurnal variation, regulated in part by nuclear receptors such as REV-ERBα that couple the molecular clock to inflammatory gene expression in macrophages (134, 138). Temporal gating of interferon and cytokine responses determines the magnitude and duration of innate activation following immunological stimulation, with direct consequences for antigen presentation and T cell priming efficiency (138).

Superimposed on circadian regulation, neuro-immune circuits exert continuous modulation of immune tone. Neural and neuroendocrine pathways can regulate antitumor immunity across multiple layers, including innate immune set points, antigen presentation, immune-cell trafficking, metabolic fitness, circadian timing, and durable immunosuppressive bias (90, 124, 139, 140). Sympathetic adrenergic signalling influences leukocyte mobilisation, endothelial permeability, and inflammatory set points, often biasing immune responses towards heightened or suppressed activation depending on neural tone. In contrast, the parasympathetic vagal axis provides fine control over cytokine production through cholinergic anti-inflammatory reflexes, restraining excessive inflammation while preserving immune competence (141, 142). These bidirectional neural pathways integrate environmental cues, stress responses, and metabolic state with immune function, thereby shaping the outcome of vaccination and subsequent checkpoint blockade.

Clinical observations provide cautious but consistent support for the relevance of these mechanisms in cancer immunotherapy (143). Retrospective analyses across multiple tumor types indicate that ICIs administered earlier in the day are associated with significantly improved progression-free and overall survival compared with late-day dosing, an effect that aligns with circadian regulation of immune trafficking and checkpoint biology (134, 144). Although these studies are observational and require prospective validation, they reinforce the principle that immune interventions operate within temporally structured systems that influence therapeutic efficacy.

Together, these data support a model in which vaccine-induced antitumor immunity is embedded within circadian and neuro-immune regulatory frameworks that modulate immune priming, amplification, and checkpoint responsiveness (139). Vaccination does not act on a neutral immune substrate, but intersects with rhythmic oscillations in immune competence and neural control of inflammation (136, 138). Consequently, identical immunological stimuli may succeed or fail depending on timing and systemic context. Recognising and integrating these temporal and neuro-immune dimensions provides a biologically grounded path towards more precise and effective immunotherapeutic strategies without invoking speculative mechanisms beyond the current evidence base (124, 139, 144).

## Design principles for next-generation vaccine–checkpoint inhibitor combinations

Tumor-agnostic vaccination strategies introduce a disruptive but increasingly evidence-based framework in cancer immunotherapy, in which the principal limiting factor is not tumor antigen availability but the systemic state of innate immune readiness (14). In this context, non-tumor vaccines function as global immune modulators rather than antigen-directed therapies, resetting host immune thresholds that condition responsiveness to immune checkpoint inhibition (39, 145, 146). Accumulating clinical and mechanistic data indicate that, in immunologically cold tumors, checkpoint blockade frequently fails not because tumors are invisible, but because the innate immune system remains insufficiently activated to license effective antigen presentation, T cell priming, and effector recruitment (20, 147).

Viral and microbial vaccines exemplify this tumor-agnostic paradigm by acting as systemic innate immune activators that induce transient, viraemia-like immune states characterised by IFN-I production, myeloid activation, and APC licensing (148, 149). Unlike tumor-specific vaccines, these platforms do not encode cancer antigens and therefore bypass constraints related to TMB, antigen heterogeneity, or HLA restriction (55). Instead, they exploit conserved innate sensing pathways to generate a permissive immune milieu in which endogenous tumor antigens, released through baseline cell turnover or therapy-induced damage, can be effectively processed and presented (55, 150). In this setting, ICIs are required to prevent compensatory inhibitory feedback, such as PD-L1 upregulation, thereby sustaining nascent antitumor T cell responses rather than initiating them *de novo* (14, 39, 147).

The most compelling clinical and translational support for this concept comes from recent analyses of SARS-CoV-2 mRNA vaccination in patients receiving immune checkpoint blockade. SARS-CoV-2 mRNA vaccines induce robust systemic innate immune activation, including interferon-α production, monocyte and dendritic-cell activation, and broad cytokine induction, effectively resetting the cancer–immunity cycle (61, 88). Retrospective analyses across large cohorts of patients with non-small-cell lung cancer and melanoma demonstrated that receipt of a COVID-19 mRNA vaccine within a defined temporal window preceding or coinciding with immune checkpoint therapy was associated with significantly improved overall survival, including in tumors lacking baseline immune infiltration (20, 88). Mechanistic studies revealed increased tumor PD-L1 expression, enhanced APC licensing and epitope spreading following vaccination, establishing that non-tumor mRNA vaccines can sensitise tumors to checkpoint blockade through systemic innate reprogramming rather than antigen targeting (20, 88).

Earlier precedents for tumor-agnostic immune activation further reinforce this model (14). BCG, one of the oldest immunotherapies in oncology, induces potent innate immune activation and has demonstrated durable efficacy in non-muscle-invasive bladder cancer. When combined with checkpoint inhibition, intravesical BCG can enhance disease control in selected contexts, although excessive or poorly constrained innate stimulation has also been associated with toxicity and limited benefit, underscoring the importance of calibrated immune activation (151–153). Similarly, oncolytic viruses function as innate immune triggers that promote interferon signalling, dendritic-cell recruitment and chemokine-mediated lymphocyte trafficking, thereby converting immune-excluded tumors into checkpoint-responsive lesions when combined with PD-1 or CTLA-4 blockade (115, 118, 154).

Despite these advantages, tumor-agnostic vaccination strategies exhibit clear and necessary limitations. Not all tumors respond to systemic innate activation, particularly those with profound stromal exclusion, defective interferon signalling, or irreversible immune suppression (16, 155). Moreover, innate immune activation alone is insufficient to sustain tumor control and requires checkpoint inhibition to prevent adaptive immune exhaustion and inhibitory feedback loops. Excessive or uncoupled inflammation also carries risks, including immune-related toxicity and non-productive immune activation, reinforcing that immune awakening must be precisely timed, dosed, and integrated with checkpoint therapy rather than maximised indiscriminately (39, 145, 146).

From a translational and regulatory perspective, tumor-agnostic vaccines offer several strategic advantages. As off-the-shelf agents, they enable rapid deployment, scalability, and broad patient eligibility independent of tumor sequencing, antigen discovery, or bespoke manufacturing pipelines. Their repurposing leverages existing safety, manufacturing, and regulatory frameworks, lowering barriers to clinical testing and accelerating combination strategies with ICIs. At the same time, their tumor-agnostic nature necessitates rigorous biomarker development to identify contexts in which systemic immune activation can be productively harnessed rather than dissipated (148, 156).

Collectively, these observations support a reframing of vaccine–checkpoint combinations in which immune activation itself becomes the therapeutic target. In selected settings, directing the immune response may be less critical than awakening it (61). By transiently resetting innate immune tone and licensing adaptive immunity, tumor-agnostic vaccines expand the conceptual and practical boundaries of cancer immunotherapy, demonstrating that effective antitumor immunity can emerge not only from antigen precision, but from systemic immune readiness appropriately aligned with checkpoint inhibition (39).

## Engineering immune readiness to overcome immunotherapy resistance

Engineering immune readiness emerges as a unifying framework to overcome the persistent limitations of immune checkpoint blockade. Across tumor types, clinical settings, and therapeutic platforms, a consistent pattern has become evident: the efficacy of checkpoint inhibition depends less on intrinsic tumor features than on the immunological state of the host at the time therapy is deployed (3, 7, 10). Tumors that respond to checkpoint blockade are not defined solely by antigenicity or mutational burden, but by pre-existing or inducible immune configurations characterised by innate activation, interferon signalling, APC competence, and permissive T cell priming environments (12, 157).

This perspective reframes primary resistance to checkpoint inhibitors as a failure of immune readiness rather than immune recognition (157). Clinical and transcriptional profiling studies have demonstrated that responses to PD-1 and CTLA-4 blockade are tightly linked to baseline interferon-driven programs, antigen presentation machinery, and chemokine gradients that enable immune cell trafficking and retention within the TME (10). Conversely, immune-cold tumors often lack these foundational signals, rendering checkpoint inhibition biologically inert even in the presence of abundant tumor antigens (3, 12, 158). These observations establish immune readiness as a rate-limiting step that precedes and conditions the therapeutic impact of checkpoint release (7).

Vaccination-enabled strategies offer a rational and mechanistically grounded means to engineer this permissive immune state. By acting upstream of inhibitory checkpoints, vaccines can initiate or amplify innate immune activation, license DCs, expand antigen-specific T cell repertoires, and synchronise adaptive immunity with checkpoint blockade (55). Importantly, the effectiveness of this approach is not dictated solely by antigen selection, but by the sequence, timing, and intensity of immune priming relative to checkpoint inhibition. Clinical evidence increasingly indicates that vaccines administered before or early during checkpoint therapy more effectively establish durable antitumor immunity than simultaneous or delayed combinations, underscoring that immunotherapy is intrinsically temporal and sequential rather than static (55, 159).

Systemic regulation further modulates this process. Interferon signalling, while essential for immune priming, can drive adaptive resistance when chronically sustained, highlighting the need for precisely calibrated immune activation rather than maximal stimulation (68, 159). Similarly, tumor-cell-intrinsic programs and stromal contexts shape how immune signals are interpreted, reinforcing that immune readiness reflects an integrated host–tumor ecosystem rather than a single molecular pathway (158–160). These insights argue against indiscriminate combinatorial escalation and instead favour deliberate engineering of immune states that are transient, productive, and checkpoint-responsive (10, 12, 68).

Crucially, this framework enables a conceptual transition away from tumor-centric targeting towards host immune state engineering (7). Rather than asking which antigens to target or which checkpoints to block, the central design question becomes how to position the immune system in a state capable of responding. Vaccines, whether tumor-directed, immune-modulatory, or tumor-agnostic, function as tools to sculpt this state, while checkpoint inhibitors serve to stabilise and sustain the responses that emerge (3). In this model, resistance is not an immutable property of the tumor, but a dynamic consequence of the immune context that can be therapeutically reshaped (55, 68, 158).

Taken together, these observations support a paradigm shift in cancer immunotherapy. Progress will not arise from adding more combinations, but from intelligently programming immune interventions in space and time. Checkpoint blockade does not fail because tumors are invisible, but because the immune system is unprepared (157). Vaccination-enabled strategies offer a rational path to engineer immune readiness and unlock durable responses in historically non-responsive cancers.

## Conclusions and future directions

Immune checkpoint blockade has irreversibly altered cancer therapy by establishing that durable tumour control can be achieved through immune modulation rather than direct cytotoxicity. However, more than a decade of clinical experience has clarified an equally important limitation: checkpoint inhibitors are not universal initiators of antitumour immunity (161–165). Their efficacy depends on a pre-existing or inducible immunological state in which antigen presentation, effector priming and tissue deployment are already underway. Primary resistance, which remains the dominant outcome across solid tumours, therefore reflects not a failure of checkpoint targeting itself, but a failure of immune readiness (10, 31, 165, 166).

This review advances immune readiness as a unifying, mechanistically grounded framework to explain why immune checkpoint blockade benefits a minority of patients while failing in most others. Across tumour types and therapeutic contexts, convergent evidence indicates that immune checkpoints function primarily as amplifiers and stabilisers of ongoing immune responses rather than as triggers of immune engagement (3, 7, 31). When innate sensing, DC licensing, antigen presentation, and lymphocyte priming have not occurred, checkpoint release is biologically inconsequential (4, 20). Cold tumours are thus not intrinsically resistant to immunotherapy; they are immunologically unprepared (167, 168).

Within this framework, therapeutic vaccination emerges not as a direct cytotoxic modality, but as a powerful upstream strategy to engineer immune readiness. Tumour-directed vaccines expand and diversify tumor-specific T cell repertoires, immune-modulatory vaccines reshape antigen presentation and inflammatory tone, and tumor-agnostic vaccines reset systemic innate activation thresholds (3, 7). Importantly, this immune-conditioning effect is not determined by antigen selection alone. Adjuvants and delivery systems are equally central because they define how antigens are interpreted by the innate immune system (35, 49). By engaging pattern-recognition receptors and inflammatory sensing pathways, adjuvants promote dendritic-cell maturation, type I interferon production, co-stimulatory signalling, cross-presentation, and lymph-node priming (50). Thus, in vaccination-enabled checkpoint therapy, antigens define what the immune system should recognize, whereas adjuvants determine whether, how, and in which inflammatory context that recognition becomes therapeutically actionable (51).

Several design principles follow from this synthesis. First, immune priming must precede or coincide with checkpoint blockade. Sequence is not a logistical detail but a mechanistic variable: unproductive checkpoint release in the absence of priming risks amplifying dysfunctional immune states rather than rescuing effective ones. Second, innate immune activation, particularly type I interferon–driven DC licensing, represents a decisive upstream bottleneck that can be therapeutically targeted. Third, adjuvant selection, dose, route, and timing should be treated as core design variables rather than secondary formulation details, because they determine the magnitude, cellular composition, duration, and spatial distribution of vaccine-induced immune readiness. Fourth, immune activation must be calibrated rather than maximised. Excessive or chronic inflammation promotes compensatory resistance, immune exhaustion and toxicity, underscoring the need for transient, programmable immune perturbations rather than sustained immune stimulation (3, 7, 29, 31, 49).

Importantly, immune readiness is not determined by tumour-intrinsic features alone, but by systemic and microenvironmental regulators that shape immune competence across space and time. The host microbiome has emerged as a key upstream determinant of basal innate immune tone, DC functionality, and interferon responsiveness (169–173). This view is consistent with the concept of host–microbiome decoupling, in which microbial signals, activities, spatial containment, or rhythmic patterns become misaligned with host regulatory capacity, thereby promoting immune miscalibration, barrier dysfunction, inflammatory instability, and altered tumour-associated processes (173). Microbiome states associated with dysbiosis can impose a systemic brake on immune priming, limiting antigen presentation and effector differentiation even in the presence of otherwise optimal immunotherapeutic interventions. Conversely, permissive microbial configurations can lower the activation threshold for vaccination and checkpoint blockade, positioning the microbiome as an actionable regulator of immune preparedness rather than a passive modifier of response (174–176).

At the tumour site, hypoxia constitutes a parallel, spatially encoded barrier to immune readiness. Hypoxic niches suppress antigen presentation, skew myeloid cells toward tolerogenic phenotypes and restrict effector T cell trafficking and function, effectively decoupling systemic immune priming from local tumour control (177–180). As a result, even robust immune activation at the systemic level may fail to translate into durable intratumoural immunity if hypoxia remains unresolved. Integrating hypoxia-aware strategies, through vascular normalisation, metabolic reprogramming or temporally coordinated immune activation, may therefore be essential to convert immune readiness into effective tumour rejection (179, 181).

Tumour-agnostic vaccination strategies further highlight the translational potential of this framework. By bypassing antigen discovery, tumour mutational burden and HLA restriction, off-the-shelf viral and microbial platforms provide scalable and regulatory-friendly means to condition immune readiness (3, 7, 182). Clinical evidence from oncolytic viruses, BCG-based therapies, and non-oncologic mRNA vaccines indicates that systemic innate immune activation can convert immunologically inert tumours into checkpoint-responsive systems. These observations expand the conceptual boundaries of cancer vaccination, reframing vaccines as immune-state engineers rather than antigen delivery vehicles (88, 115, 151, 183, 184).

Future progress will depend on translating this conceptual clarity into deliberate clinical design. Trials should be structured around immune state transitions rather than static treatment arms, with immune readiness treated as a measurable and programmable variable (3, 5). Biomarker development should prioritise functional immune competence, innate activation, DC abundance, interferon signalling, microbiome configuration, and trafficking capacity, rather than tumour-centric features alone (12, 20, 185, 186). Therapeutic success will require not more combinations, but better choreography: aligning vaccination, checkpoint inhibition, and host immune dynamics in space and time (4, 187).

In summary, checkpoint blockade does not fail because tumours are invisible, but because the immune system is unprepared. Engineering immune readiness, through vaccination, innate reprogramming, microbiome modulation, and temporal immuno-engineering, provides a coherent strategy to overcome primary resistance and expand the therapeutic reach of immunotherapy. The next phase of cancer immunotherapy will not be defined by new checkpoints alone, but by our ability to deliberately construct the immune conditions under which those checkpoints matter (7, 31, 112, 186).
